# Brain health services for the secondary prevention of cognitive impairment and dementia: Opportunities, challenges, and the business case for existing and future facilities

**DOI:** 10.1016/j.tjpad.2025.100098

**Published:** 2025-03-17

**Authors:** Giovanni B. Frisoni, Federica Ribaldi, Gilles Allali, Théophile Bieth, Andrea Brioschi Guevara, Stefano Cappa, Lisa Cipolotti, Kristian Steen Frederiksen, Jean Georges, Frank Jessen, Giacomo Koch, Hugh Masters, Augusto J. Mendes, Lutz Frölich, Valentina Garibotto, Oriol Grau-Rivera, Federico E. Pozzi, Dorota Religa, Ayda Rostamzadeh, Lenny Shallcross, Susan D. Shenkin, Wiesje M. van der Flier, Meike W. Vernooij, Leonie N.C. Visser, Jeffrey L. Cummings, Philip Scheltens, Bruno Dubois, Elena Moro, Claudio L.A. Bassetti, Miia Kivipelto

**Affiliations:** a Memory Center, Department of Rehabilitation and Geriatrics, University Hospitals and University of Geneva, Geneva, Switzerland; b Leenaards Memory Centre, Department of Clinical Neurosciences, University Hospital of Lausanne (CHUV), Lausanne, Switzerland; c Institut de la Mémoire et de la Maladie d'Alzheimer, IM2A, Groupe Hospitalier Pitié-Salpêtrière, Sorbonne Université, Paris, France; d Faculty of Psychology and Educational Sciences, University of Geneva, Geneva, Switzerland; e Federation of the European Societies of Neuropsychology (FESN) University Institute of Advanced Studies Pavia, Italy; f IRCCS Istituto Auxologico Italiano, Milan; g Federation of the European Societies of Neuropsychology (FESN), Switzerland; h European Academy of Neurology (EAN), Switzerland; i Alzheimer Europe, Switzerland; j Department of Psychiatry, Faculty of Medicine and University Hospital Cologne, University of Cologne, Cologne, Germany; k German Center for Neurodegenerative Diseases (DZNE), Bonn-Cologne, Germany; l Excellence Cluster Cellular Stress Responses in Aging-Related Diseases (CECAD), Medical Faculty, University of Cologne, Germany; m Department of Clinical and Behavioural Neurology, Santa Lucia Foundation IRCCS, 00179, Rome, Italy; n Department of Neuroscience and Rehabilitation, University of Ferrara, Ferrara, Italy; o Center for Translational Neurophysiology of Speech and Communication (CTNSC), Italian Institute of Technology (IIT), Ferrara, Italy; p Brain Health Scotland United Kingdom; q European Alzheimer's Disease Consortium, Switzerland; r European Association of Nuclear Medicine (EANM), Switzerland; s Barcelonaβeta Brain Research Center (BBRC), Pasqual Maragall Foundation, Barcelona, Spain; t Clinica Neurologica, IRCCS San Gerardo dei Tintori, Monza, Italy; u European geriatric medicine society (EuGMS), Switzerland; v Karolinska Institutet, Stockholm, Sweden; w World Dementia Forum, London, UK; x University of Edinburgh, Edinburgh, Scotland United Kingdom; y Alzheimer Center Amsterdam, Department of Neurology, Amsterdam Neuroscience, Amsterdam UMC, Amsterdam, The Netherlands; z Amsterdam Neuroscience, Neurodegeneration, Amsterdam, The Netherlands; aa Epidemiology and Data Science, Vrije Universiteit Amsterdam, Amsterdam UMC location VUmc, Amsterdam, The Netherlands; ab European Society of Neuroradiology (ESNR), Switzerland; ac Division of Clinical Geriatrics, Center for Alzheimer Research, Department of Neurobiology, Care Sciences and Society, Karolinska Institutet, Stockholm, Sweden; ad Department of Medical Psychology, Amsterdam Public Health Research Institute, Amsterdam UMC, Amsterdam, The Netherlands; ae Chambers-Grundy Center for Transformative Neuroscience, Department of Brain Health, School of Integrated Health Sciences, University of Nevada, Las Vegas, NV, USA; af EQT Life Sciences, Amsterdam, The Netherlands; ag Institut du Cerveau et de la Moelle Épinière, UMR-S975, INSERM, Paris, France Hôpital de la Pitié-Salpêtrière, France; ah European Brain Council, European Academy of Neurology, Swiss Brain Health Plan, Switzerland; ai Inselspital and Faculty of Medicine, University of Bern, Bern, Switzerland; aj Institute of Public Health and Clinical Nutrition, University of Eastern Finland, Kuopio, Finland; ak Theme Inflammation and Aging, Karolinska University Hospital, Stockholm, Sweden; al The Ageing Epidemiology Research Unit, School of Public Health, Imperial College London, London, UK

**Keywords:** Brain health services (dBHS), Dementia, Secondary prevention, Preventive healthcare, Public health

## Abstract

A European Task Force has recently developed and published the concept and protocols for the setup of the innovative health offer of Brain Health Services for the secondary prevention of dementia and cognitive impairment (dBHS). dBHS are outpatient health care facilities where adult persons can find an assessment of their risk of developing cognitive impairment and dementia, have their risk level and contributing factors communicated using appropriate language supported by adequate communication tools, can decide to participate to programs for personalized risk reduction if at higher risk, and benefit from cognitive enhancement interventions. This health offer is distinct from that of currently active memory clinics. The ultimate aim of dBHS is to extend healthy life, free from cognitive impairment. Here, we (i) discuss the pertinent opportunities and challenges for those persons who want to benefit from dBHS, professionals, and wider society, (ii) describe the concepts, protocols, organizational features, and patient journeys of some currently active dBHS in Europe, and (iii) argue in favor of the business case for dBHS in Europe.

## 1 Introduction

The notion that dementia can be prevented or postponed has recently gained credit in the community of experts. Population-based epidemiological studies have consistently shown a progressively reduced risk of dementia in Western society and, more specifically, Alzheimer's dementia at any age in the past four decades, possibly related to healthier lifestyles and better control of vascular risk factors [1]. Structured multi-domain interventions consisting of diet, exercise, cognitive training and social stimulation, sleep control, and vascular risk monitoring has shown better cognitive outcomes, particularly but not only in persons at high vascular risk [2,3]. Although the debate is lively on clinical meaningfulness, anti-amyloid drugs have shown some efficacy at slowing progression in patients with Alzheimer's disease in the earliest cognitive impairment stages, laying the theoretical foundations for secondary prevention in the asymptomatic at risk [4]. Recent reports suggest that it is possible to induce long-lasting improvement of cognitive performance in cognitively unimpaired older persons with non-invasive brain stimulation [5].

We believe that preventing cognitive impairment in unimpaired people at risk of cognitive decline and dementia requires specific skills and protocols that differ from those employed in current memory clinics. In a previous publication, we have described what these new skills and protocols consist of and have argued that they take the form of a new patient journey, that may be offered in dedicated clinical services that we called Brain Health Services for the Prevention of Cognitive Impairment and Dementia (dBHS for short) [6]. Activities in dBHS consist of the four pillars of assessment of the risk of developing cognitive impairment/dementia, specific techniques and tools for the communication of that risk, personalized risk reduction programs for those persons at higher risk and offer cognitive enhancement interventions [6].

As is the case of any innovation, dBHS present with challenges as well as opportunities. Some of these are specific to the four activity pillars mentioned above. Others are horizontal to all pillars such as organization and leadership, education, stakeholder engagement, ethics and use of resources. The latter is of relevance at the current early development stage where activities are not reimbursed by health care systems and insurances.

This paper summarizes the discussions held at the International Conference and Workshop on Brain Health Services for the Prevention of Dementia, on February 8, 2024, in Geneva, Switzerland. It discusses opportunities and challenges for service users, professionals, and wider society, offers a state-of-the-art of some current dBHS pilot experiences, and finally addresses the business case for the secondary prevention of dementia and Alzheimer's disease in dBHS. The coauthors of this paper encompass representatives from the major stakeholders, i.e. members of the task force which developed the dBHS concept, early professional adopters, pertinent scientific societies, disease-based professional networks, pharma industry, private research funders, patient advocates, charities and international organizations. Recommendations to centers aiming to establish their own dBHS have been provided in previous publications [[6], [7], [8], [9], [10], [11], [12], [13]], to which the present is a follow-up.

## 2 Opportunities and challenges of dBHS

Risk assessment will encompass the twelve modifiable risk factors identified by The Lancet Commission in 2020 [14], apolipoprotein E (APOE) genotyping, and assessment of biological indicators of brain disease such as brain beta-amyloid, brain tauopathy, neurodegeneration, and cerebrovascular disease. More factors may be added to the list as evidence accrues (e.g. sleep disturbances [15], polypharmacy, diet [16], alpha-synuclein, synaptic density, to name a few) [17,18].

Ultra-sensitive assays are already commercially available that will allow upscaling of the measurement of biomarkers of neurodegeneration and Alzheimer's pathology [19] and expand to other biomarkers such as neurofilament light (Nfl) and glial fibrillary acidic protein (GFAP). Risk communication will benefit from apps allowing to draft individualized infographics on the fly by entering the person's risk factors and visualizing their cumulative risk [20]. Some risk reduction programs are available focusing mainly on the cerebrovascular component of cognitive impairment, such as the Finnish Geriatric Intervention Study to Prevent Cognitive Impairment and Disability (FINGER) protocol [2]. Although it might be argued that the cognitive training and physical activity components of the FINGER intervention may have effects beyond vascular pathways, there is limited evidence to date that risk reduction interventions focusing solely on the neurodegenerative component (with e.g. anti-amyloid drugs and synaptic plasticity enhancers such as multicomponent nutritional supplements) [20,21] translate to a reduction in clinically meaningful outcomes of cognitive function or dementia incidence in cognitively normal individuals. However, the use of biomarkers as surrogate endpoints for regulatory approval of anti-amyloid monoclonal antibodies in prodromal to mild Alzheimer's disease provides an interesting working framework for secondary prevention in the unimpaired at risk. Although conceptually distinct, cognitive enhancement (or neuroenhancement) will often accompany risk reduction and use non-invasive brain stimulation techniques (NIBS, with electrical or magnetic stimuli) and behavioral cognitive training.

Opportunities and challenges arise for a number of stakeholders as dBHS transition from research into clinical services. Stakeholders and their delegates at the International Conference and Workshop are listed in the pertinent section at the end of the manuscript. Table 1 shows opportunities and challenges from the viewpoint of pertinent scientific societies, disease-based professional networks, pharma industry, private research funders, patient advocates, and charities and international organizations.

**Table 1:** Opportunities and challenges associated with the foundational pillars of dBHS and enabling factors.

THE FOUR PILLARSRisk assessment • Promoting large population-based studies with long follow-up to estimate the adjusted risk of lifestyles, genetics, and imaging and fluid biomarker risk factors • Implementing sex-, gender-, ethnicity-specific risk assessment and management • Promoting comprehensive physical health assessment • Promoting assessment of sleep habits and sleep loss/disorders • Management of incidental MRI findings • Shortage of trained health care workers • Implementing novel blood biomarkers for neurodegenerative diseases and stremaline them with traditional CSF and PET biomarkers • Radiation exposure for serial PET molecular biomarker assessment • AI for individualized risk profiling Risk communication • Tailoring risk communication according to socio-cultural factors • Development and validation of operator-free digital risk communication tools • Management of negative psychological responses to risk disclosure Personalized prevention • Interventions based on biomarker features • Identification of patient-oriented outcomes • Programs for those developing incident cognitive impairment and dementia despite prevention • Integrated pharma and non-pharma risk reduction programmes • dBHS as a platform for outcome and cost-effectiveness research Cognitive enhancement • Finetune non-invasive brain stimulation protocols for maximal efficacy in cognitively unimpaired • Promote tailored multimodal interventions (non-invasive brain stimulation with traditional/digital cognitive interventions and pharmacologic enhancers) • Promote social engagement through group activities • Ensure equality of access to highly technological interventions • Need of trained personnel and infrastructure for non-invasive brain stimulation
ENABLERSOrganization and leadership • Clear definition of where lies innovation in Brain health • Seamless integration of dBHS into current health care provision • Technological investment on digitalization • Competition between current tertiary and future secondary prevention • Development of locally adapted models of health care provision and reimbursement for dBHS Education • Dissemination of standardized and optimized cognitive and imaging acquisition protocols • Educational programmes on brain health in academia (e.g. European Master Course on Brain Health of the European Academy of Neurology) • Education of GPs on cognitive screening and post-screening patient management • Stakeholder engagement • dBHS as natural laboratories for new models of multidisciplinary collaboration • dBHS as flagship initiative of the European Academy of Neurology and the Swiss Federation of Clinical Neuro Societies • Leverage on current continental inter-societal collaborations led by scientific societies • dBHS as a space for cooperation between neurology and mental health • dBHS contribute to current WHO priorities Ethics and resources • Leverage on prevention to de-stigmatize dementia • Prevent ageism bias inherent in secondary prevention programs • Ensure equality of access in an era of expensive biomarker assessment technology • Ensure a balanced distribution of tasks and resources between dBHS and primary care • Prevent the resource drain from tertiary to feed secondary prevention • Ensure secondary prevention even to community with limited resources and greater need for immediate tertiary prevention

Opportunities and challenges are addressed separately for the four foundational pillars of dBHS, (i.e. risk assessment, risk communication, personalized risk reduction, and cognitive enhancement) and for enabling factors horizontal to all pillars, i.e. organization and leadership, education, stakeholder engagement, and ethics and use of resources. We acknowledge that most opportunities to exploit dBHS will come at the cost of challenges to overcome, and the following text will not try to artificially categorize them.

### 2.1 The four pillars

#### 2.1.1 Risk assessment

Risk factors for dementia are related to lifestyles, genetics, and imaging and fluid biomarkers [6,14]. Risk estimates for lifestyle risk factors are based on accurate population-based studies that only exceptionally consider genetic, imaging, and fluid biomarkers, while the latter are accurately studied in convenience cohorts not representative of the general population and in general disregard lifestyles. Future studies will need to estimate the risk for cognitive impairment and dementia in representative population-based cohorts with long follow-up where lifestyles, genetics, and imaging and fluid biomarker risk factors are equally and accurately studied at baseline.

Risk factors encompass medical, biological, psychological, functional, physiological, social, and environmental dimensions. An assessment of the risk for dementia provides an opportunity to promote Multi-dimensional Geriatric Assessment, a conceptual framework of care developed for older patients taking into account medical, psychological, functional, social and environmental dimensions of health, which could be beneficial to patients at all ages, and in diverse settings (community and hospital) [22,23].

One of the risk factors for dementia is neurodegenerative changes (i.e. atrophy) on magnetic resonance imaging (MRI) [6]. Studying asymptomatic persons with brain MRI may lead to incidental findings, with about one in 27 requiring follow-up [24] and dBHS will need to develop clear protocols to manage such [[25], [26], [27]]. Blood-based biomarkers for Alzheimer pathology, once implemented in the real world [28] will allow large-scale screening programs and risk estimation in many persons. However, they may be insufficient in some cases and not be able to offer the accuracy of topographic biomarkers such as tau positron emission tomography (PET) [29]. Serial tau PET over time may be indicated in the future to detect pathological progression in a minority of very high-risk candidates for anti-amyloid treatment, with the consequent radiation exposure of an asymptomatic population. Current technological developments on high-sensitivity systems will need to be leveraged to reduce the costs of tracers and the exposure associated with each procedure and thus the impact of repeated investigations.

Risk assessment in dBHS may lead to an increased number of brain MRI and PET/Single-photon emission computed tomography (SPECT) scans, and cognitive assessments, leading to a relative shortage of imaging facilities and psychologists, neuropsychologists, neuroradiologists, and nuclear medicine physicians [30,31]. High-level programming of university degree output will need to take these new needs into account.

Recent advancements in Artificial intelligence (AI) applied to neuroimaging based on multivariate brain network features from one or more neuroimaging modalities (the “predictome”) [32] have shown promising outcomes for individualized characterization of persons at risk of developing cognitive impairment and dementia [33]. The dBHS initiative is in line with this trend of moving from detecting disease to assessing health status and disease risk.

#### 2.1.2 Risk communication

Communicating risk is a complex exercise even to mathematically literate persons. The recommendations we have developed for risk communication [6,10] will need to be adapted and validated to all levels of mathematical literacy.

Both the collection and the communication of the risk of cognitive impairment and dementia is currently done largely by human operators. Operator-free digital risk assessment and communication tools will need to be developed to upscale risk assessment and communication and decrease the costs of large prevention programs in the population.

Our recommendations for risk communication place strong emphasis on the management of negative psychological responses to risk disclosure [6,10]. They will need to be adopted and validated on psychological outcomes in real-life conditions across different settings, ethnicities, cultures, and educational backgrounds.

#### 2.1.3 Personalized prevention

Secondary prevention of dementia should head strongly towards the fine-grained selection of at-risk persons based on genetic or biomarker features (e.g. biomarkers of amyloid or tau pathology), with the aim of maximizing intervention effectiveness and thus enhancing the cost/benefit ratio. For instance, the FINGER interventions have been shown particularly effective in APOE4 carriers [34]. This has been replicated in Japan [35].

Prevention programs are by nature long-term. They thus require expensive trials to detect effectiveness on clinical outcomes. The validity of surrogate outcomes should be investigated to improve feasibility and reduce costs. Feasible and meaningful patient-oriented outcomes need to be identified to estimate the clinical impact of dBHS and their comparative cost-effectiveness.

Prevention programs will not reduce to nil the incidence of the condition of interest. Participants developing cognitive impairment and dementia despite participation to risk reduction programs run the risk of stigma and blame. There should be clear pathways to appropriate medical and psycho-social support in health and social care systems for appropriate prevention of such adverse outcomes [36].

Multiple risk factors for dementia are often present in the same person. For instance, brain amyloidosis may come with social isolation, depression, and alcohol abuse. Sleep disorders are frequently associated with depression, alcohol abuse, medical/neurological comorbidities and physical inactivity. Integrated programs will need to be developed that will maximize the benefit on risk reduction by anti-amyloid medications [37,38] and associated lifestyle interventions [39]. Until their efficacy for prevention is demonstrated, dBHS are a gateway to prevention trials.

#### 2.1.4 Cognitive enhancement and neurostimulation

Cognitive enhancement is a critical pillar of dBHS, addressing patient expectations while complementing the other pillars. Indeed, anecdotal evidence from dBHS pilot centers highlights that SCD individuals often seek more than just knowledge of their risk and strategies to reduce it; they express a strong desire for interventions aimed at improving cognitive performance. This observation is supported by Harrell et al. [40], who emphasize the motivation of individuals to engage in cognitive training programs to enhance cognitive function.

NIBS is one of the most promising techniques for cognitive enhancement and possibly risk reduction [41]. While NIBS protocols are often currently being tested in single-intervention mode, the dBHS concept paves the way to the evaluation and implementation of NIBS in multimodal brain health programs in which advanced-brain stimulation protocols are associated with cognitive interventions in the context of risk reduction programs [42].

Novel methodologies allowing tailored neurostimulation combined with digital cognitive interventions can foster high-quality studies of cognitive training/stimulation and neuromodulation [43]. Stand-alone digital interventions for cognitive symptoms in people without dementia are a fast-growing field [44] and their comparative efficacy with combined interventions should be elucidated.

### 2.2 Enabling factors

#### 2.2.1 Organization and leadership

When communicating to decision makers, the concept of brain health should be used sparingly. Care should be exercised not to recycle old concepts with new labels, which will jeopardize the credibility of the brain health construct. Indeed, the boundary between the innovative edge of the brain health concept, and current neurological practice can be fuzzy. Prevention can be primary, secondary, tertiary, and quaternary, and a large share of medical acts fall under this large umbrella. Few national programs have been launched (e.g. in Norway, Germany and Switzerland) with a combined holistic and personalized approach to promote brain health and prevention of neurological/brain disorders [[45], [46], [47]]. Proponents of so-called innovative brain health programs should clearly define where innovation lies. In the dementia field, this consists in secondary prevention of cognitive impairment and dementia in cognitively intact persons.

The emphasis on lifestyle risk factor interventions may lead health care providers and industry to perceive dBHS as competitors to traditional memory clinics and monoclonal antibody treatments. The message should be clearly spelled out that dBHS and traditional memory clinics are conceptually and practically distinct, address different patient populations, offer distinct medical services, and are not alternative but rather complementary. dBHS will need to seamlessly integrate into the health care network of both current specialist care (in neurology, psychiatry, geriatrics, neuropsychology as well as traditional memory clinics), and primary care/general practice. The setup of yet another healthcare silo should be avoided. A hub-and-spoke model for integration has been proposed in the context of the Swiss Brain Health Plans, awaiting implementation [45].

Substantial technological investment needs to be done on digital infrastructures for the efficient management of neuropsychological assessment, including remote and unsupervised data collection [48]; storage and analysis of behavioral data; behavioral training for cognitive rehabilitation; and integration of digital data into the health care network [49].

Diverse models of health care provision and reimbursement co-exist in European countries such as universal pro-capita public system coverage, and universal pro-capita private insurance coverage, social health insurance, and mixed systems. Some potential business models have been outlined in a previous publication [8]. However, a one-size-fits-all approach will not be applicable to integrating the dBHS concept in clinical practice, and country-specific solutions will need to be devised.

#### 2.2.2 Education

Education on brain health and the related concepts and lexicon should touch first and foremost the prospective users of dBHS, i.e. virtually all citizens without cognitive impairment above the age of 50–60 years, and society at large. Scientific societies and patient organization should ally to develop awareness around brain health in general and cognitive health in particular. The example of the Brain Strategy of the European Academy of Neurology (see section on Stakeholder engagement) should be expanded to a larger number of institutional stakeholder and grassroot organizations [45].

Education on brain health is absent not only in the society, but also in academia. The first world-wide educational program on brain health, a certificate of advanced studies (CAS), open to MD's but also other health professional will be launched in fall 2024 by the University of Bern with the support of the Swiss Brain Health Plan, the Swiss Federation of Clinical Neuro-Societies, the European Academy of Neurology, and other organizations [50]. More effort should be devoted to promoting post-graduate education on brain health by other and diverse stakeholders.

Generalists including general practitioners (GPs) should receive clear guidance on the administration and interpretation of cognitive screening tests. They should also be educated to refer patients screening positive (i.e. with cognitive impairment) to memory clinics, and negative (i.e. with no cognitive impairment) at potentially high risk to dBHS. In so doing, they should not rely only on the results of the screening test but also evaluate the patient request on the basis of the global clinical and psycho-social history. Emphasis on prevention should not reduce the education and support to GPs on the management of older people diagnosed with dementia.

Neuropsychological test norms are severely biased towards persons of white ethnicity. Many widely used tests do not take advances in cognitive neuroscience into account and have been normed in non-contemporary cohorts of control subjects. Greater emphasis will need to be placed on longitudinal changes at the individual level, rather than on cross-sectional reference to normative values. Neuropsychologists will need to be educated in the assessment of different racial/ethnic groups and minorities, requiring the cultural and linguistic adaptation of assessment and rehabilitation tools [51].

Digital tools for neuropsychological assessment will play a key role in future prevention programs, for their capability of fast and frequent assessment of cognitive status. A number of reviews are available on this topic [[52], [53], [54]]. The longitudinal assessment of cognitive performance reduces the reliance on normative data, which may be inappropriate for the evaluation of cognitive decline in individual subjects. In addition, digital tools offer the possibility of low-cost web-based remote assessment and can generate large datasets for AI-based data analysis.

Most patients undergoing MRI and PET for cognitive complaints are scanned with suboptimal protocols and images are read with the traditional visual rating only [55,56]. Neuroradiologists and nuclear medicine physicians will need to be educated to the use of the appropriate MRI acquisition sequences and protocols [55], semi-quantitative visual rating scales, and automated quantitative image post-processing tools [57,58].

#### 2.2.3 Stakeholder engagement

The first stakeholders of dBHS are their users. The ongoing pilot experiences should integrate the evaluation of user expectations and acceptability of the dBHS approaches [59].

The dBHS concept will need multi-disciplinary expertise to be implemented in the clinic and will be natural laboratories to develop new models of multidisciplinary collaboration. In addition to medical specialists such as neurologists, geriatricians, old-age psychiatrists, neuroradiologists, nuclear medics, laboratory medics, geneticists, epidemiologists, ear nose and throat specialists, dentists, diabetes specialists, and specialists in primary care, non-medical professions will be required such as psychologists, neuropsychologists, nurses, physiotherapists, occupational therapists, nutritionists, and speech and language therapists. dBHS might be flagship initiatives for scientific societies. The European Academy of Neurology has publicly engaged into the brain health space and may be a worldwide leader and forerunner in the development of dBHS. It has recently launched the Brain Health Strategy under the motto of “One brain, one life, one approach” with the aim of raising awareness on brain health and fostering related initiatives by building a brain health alliance, supporting international and national/regional policy making, promoting research and, education, and raising public awareness & understanding [50]. The Swiss arm of the European Brain Health Strategy is being built under the auspices of the Swiss Federation of Clinical Neuro Societies [45], where the dementia pillar is based in Geneva, and being developed along the lines defined by the dBHS.

Risk reduction of cognitive impairment and dementia should find a space which is at the same time inclusive and non-conflicting with mental health and building a world-wide alliance outside the natural geographical boundaries of committed stakeholders such as the European Academy of Neurology. The current borders delineating neurology and psychiatry will have less relevance regarding brain health and prevention of dementia and mental disorders. A holistic approach will view brain health as a whole and indivisible space where all specialties can and should contribute. Synergies should be sought between pilot experiences and current large European research projects on screening and prevention such as EPAD - European prevention of Alzheimer's dementia [21], PREDICTOM – Prediction of Alzheimer's disease using an AI driven screening platform [60], AD-RIDDLE – Real-World Implementation, Deployment, and Validation of Early Detection Tools and Lifestyle Enhancement [28], world-wide FINGER [61].

Importantly, dBHS contribute to the current World Health Organization (WHO) priorities on a life-course approach to aging, where addressing individual's needs at all ages (i.e. earlier in life) aims to safeguard their human right to health throughout their lifetime [16].

#### 2.2.4 Ethics and resources

Dementia is still a highly stigmatized condition, and the ethical implications of BHS and secondary prevention programmes has been addressed in a previous publication from our group [13]. Societal awareness that it can be prevented, treated and supported might contribute to de-stigmatize it, as has been the case for cancer and some psychiatric disorders. Consideration should be given to the communicative power of nomenclature: the lengthy label “Brain Health Services for the secondary prevention of cognitive impairment and dementia” may need being revisited as well as the dBHS acronym.

Prevention programs are more cost-effective in those populations with longer life expectancy allowing to reap the benefits of better health over a longer time span. dBHS proponents should be careful to prevent discrimination of their interventions based on age but at the same time maximize the benefit of their intervention for the cumulative health of society. dBHS require a significant amount of expensive diagnostic technology for risk assessment such as blood, genetic markers, and possibly in some cases and MRI, cerebrospinal fluid (CSF) biomarkers, and PET. Proponents of dBHS will need to ensure that the design of services do not translate into unequal access.

Screening programs can leverage on existing prevention programs to optimize resource allocation. For instance, if dBHS discover high blood pressure or high cholesterol, the individual may be redirected to their GP for treatment and follow-up. The development of effective coordination and synergies with primary care/GPs will be paramount to optimize resource use across health systems.

Resources should not be drained from tertiary to feed secondary prevention, i.e. from those with cognitive impairment and dementia to the unimpaired. Developing solutions to prevent dementia will not decrease the prevalence of dementia overnight, and resources will always be needed – and hopefully increased – to treat persons with cognitive impairment and dementia. dBHS proponents should ensure they are not perceived as diverting resources from people with immediate health needs.

Investing resources in secondary prevention can be challenging to communities with compelling immediate health care needs and limited resources such as the oldest old, those with comorbidities including mental health issues, functional or sensory impairment, reduced mobility, lower socioeconomic status, ethnic minorities, socioeconomic deprivation, lower health literacy, and migrants. Equitable access will need to be assured to preventative services while maintaining provision of health care services.

## 3 Currently active dBHS in Europe

We describe structure and activities of eight European pilot dBHS based in Aberdeen, Scotland (UK); Amsterdam, The Netherlands; Barcelona, Spain; Cologne, Germany; Geneva, Switzerland; Monza, Italy [62]; Paris, France; and Stockholm, Sweden. These have been identified largely through personal knowledge as the literature was unhelpful. A PubMed search with “(service[ti] OR clinic[ti]) AND prevention[ti] AND (dementia[ti] OR alzheimer*[ti])” last run on May 4, 2024 retrieved just five items, four of which were non pertinent and one referred to a in memory service in New York, USA [63]. The resulting list does not claim to be a thorough inventory of all existing pertinent initiatives in Europe, but a convenience sample from reputed academic memory clinics. All but two (Barcelona and Cologne) have been developed as extensions of traditional memory clinics. The search was limited to Europe for the remarkably different structure of health care in other country, notable the US.

The major operational difference among these pilot dBHS is related to the clinical or research nature of the patient journey. In five dBHS (Aberdeen, Geneva, Monza, Paris, and Stockholm), the patient journey is part of clinical activities or hybrid clinical and research, while in the remaining it is framed solely in the context of funded research projects with pertinent ethical clearance. These dBHS are traditional memory clinics which developed patient journeys by piecing together bits of own and literature scientific evidence. Among these, those relatively less developed journeys are offered in the context of existing health care reimbursement schemes, while those more developed are funded through a hybrid health care reimbursement and research project funding scheme. dBHS with purely research-based patient journeys are set in the context of either traditional memory clinics which have put in place one or more research projects addressing the issues pertinent to the development of a secondary prevention patient journey (Amsterdam and Cologne) or a purely academic research center with no clinical mission (Barcelona).

### 3.1 Patient journeys

**Fig. 1:**
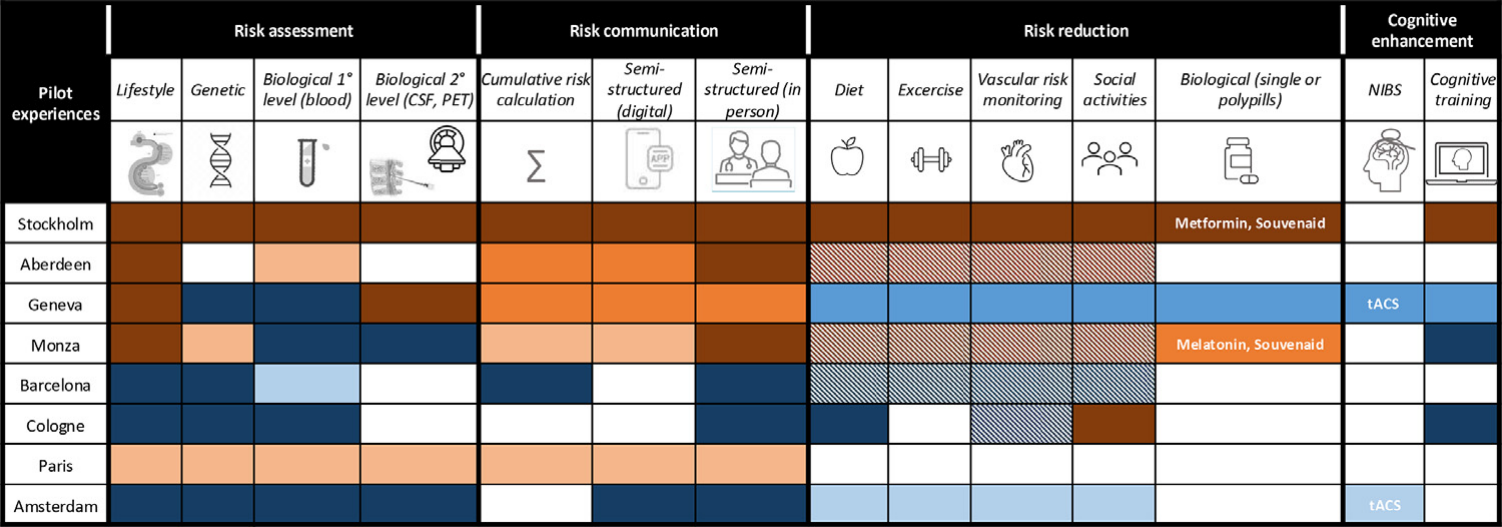
Patient journeys in eight pilot dBHS in Europe. The first three rows of the spreadsheet illustrate the ideal patient journey in a dBHS, detailing the primary activities within each of the four pillars: risk assessment, risk communication, risk reduction, and cognitive enhancement. Pilot dBHS are ordered from the most clinically oriented to the most research oriented. Brown shades  denote the clinical setting, blue shades  denote research projects. The stage of development is denoted as follows: dark shades  are ongoing activities, medium shades  are activities under development, light shades  are activities being planned. Cells with diagonal lines  indicate that advice and referral to other specialists is provided to users rather than inclusion into structured programs.

The patient journey covering all the activities pertinent to a dBHS (risk assessment, risk communication, risk reduction, and cognitive enhancement), was outlined in 2023 by the European Task Force for Brain Health Services for the Prevention of and Cognitive Impairment and Dementia [6]. Fig. 1 illustrates the current state of implementation of the four pillars of the ideal dBHS patient journey across pilot experiences.

*Risk assessment*. Alongside the twelve lifestyle risk factors originally identified by Livingston et al. (2020) [14], many pilot dBHS collect information on visual impairment, cholesterol levels, sleep quality, and dietary habits. Biological risk factors such as amyloid and tau pathology are frequently assessed using blood biomarkers (1st level assessment), although this remains primarily within the scope of research activities. While the polygenic risk score has some evidence for risk prediction, its implementation in dBHS has not yet occurred. Conversely, APOE genotyping continues to be the gold standard for genetic risk assessment, although again still in research projects.

Lifestyle risk factors are integrated into the clinical settings across all pilot experiences, with genetic risk factors increasingly integrated into patient journeys for both clinical settings and research projects. The adoption of biological risk assessments through blood-based biomarkers or tau PET remains mostly confined to research projects. In contrast, amyloid PET or CSF analysis (2nd level assessment) have been integrated into the clinical activities of half of the pilot experiences. Cardiovascular Risk Factors, Aging and Dementia (CAIDE) and Combinostics Disease State index are used in Stockholm to compute the cumulative risk [[64], [65], [66]].

*Risk communication*. We will not reiterate here the recommendations previously developed by the Task Force [6]. Pilot dBHS place particular emphasis on the emotional status of the patient during risk communication, and the availability of a safe environment for the expression of emotions. As suggested by the Task Force, visual aids and plain language were enforced, with an emphasis on the two-way exchange of information, including a conversation on patients' motivations and needs, and the teach-back technique to check understanding.

Significant effort is being devoted developing new digital tools and applications to assist in the communication of absolute and cumulative risk. At the moment, these are not part of a clinical journey and only used in the context of research projects. In contrast, the semi-structured in-person disclosure of dementia risk [10] is becoming increasingly common.

*Risk reduction*. The risk reduction pillar is marked by advancements, particularly in lifestyle interventions. Stockholm has developed and implemented the FINGER activities in clinical practice, with most pilot experiences following suit, either through structured lifestyle programs in research projects or advice in clinical settings. The FINGER study has served as an inspiration for recognizing the effectiveness of lifestyle and vascular interventions. However, these interventions still face barriers in being used in clinical settings due to incomplete reimbursement and the need for specialized personnel. In Stockholm, the Metformin (MET)-FINGER study is integrating a FINGER lifestyle intervention, targeting primarily vascular risk, with metformin, targeting multiple metabolic and neurodegenerative pathways [67]. Fortasyn connect (Souvenaid) is used in two dBHS based on the 24-month intervention with a specific multinutrient in people with prodromal Alzheimer's disease (LipiDiDiet) and Multimodal Preventive Trial for Alzheimer's Disease (MIND-AD) trials [68,69]. Stockholm is also testing a digital FINGER intervention related to stress management and sleep in a specific research project [70].

*Cognitive enhancement*. NIBS falls largely under research projects. This is not surprising given that current evidence supporting the efficacy of NIBS in cognitively unimpaired individuals remains sparse. In contrast, four out of the eight pilot experiences assessed are currently incorporating cognitive training into their protocols, either within research environments or clinical practice. Notably, cognitive enhancing drugs are not reportedly in use in any of the dBHS, in agreement with the results of a previous review from the Task Force [12].

## 4 The business case for brain health services for the prevention of dementia

Dementia disorders are among the costliest conditions, both per affected individual as well as in terms of total socioeconomic impact [71]. The worldwide cost has been estimated to $1.3 trillion annually and is bound to increase in coming years with the escalating prevalence of dementia disorders due to ageing populations. A recent study on burden of neurological disorders ranked dementia disorders as the third largest contributor to lost disability-adjusted life years world-wide [72]. Developing strategies to prevent the onset and progression of cognitive impairment is a health policy priority [73].

The four pillar activities in dBHS have the goal of preventing or postponing the onset of cognitive decline and dementia [6]. The introduction of this novel concept in health care systems raises the question of whether dBHS represent good value for money, and how such programs can be designed to be cost-effective [8]. The incremental cost-effectiveness ratio for a health care program is calculated as the estimated change in cost divided by the change in effectiveness, often quantified as the number of quality-adjusted life-years (QALYs) gained, by introducing the program, compared to the current standard of care [74]. A QALY is a ‘common currency for health’, encompassing mortality effects as well as changes in quality of life, and allows programs in different disease areas to be compared; one QALY is equivalent to one year of perfect health.

### 4.1 Model and assumptions

The potential cost-effectiveness of dementia prevention can be modeled based on data on baseline dementia risk, hypothesized effect size, costs and utility losses associated with dementia health states. To provide illustrative and preliminary estimates of the potential value gained with dBHS, we have adapted a previous model developed to assess the cost-effectiveness of the FINGER program [75]. The model consists of five health states: at risk of developing dementia, mild, moderate and severe dementia, and death. We have updated the model with cost data by European region from a systematic review and meta-analysis [76]. We have assumed that the program will result in a 5-year reduction in the risk of dementia by 20%, acknowledging that this assumption is illustrative rather than definitive. While possibly optimistic, this figure aligns with outcomes observed in controlled trials. Disease-modifying antibody therapies have demonstrated reductions in Alzheimer's disease progression around 25–30%, and the FINGER trial showed a 23% reduction in cognitive decline over 24 months through multi-domain lifestyle interventions in high-risk populations [77]. The assumption provides a reasonable baseline for hypothetical modeling while highlighting the potential variability in real-world outcomes.

We have estimated the changes in time spent in dementia states and the effect on mortality if the intervention is initiated at different ages between 60 and 85. Further, we have conservatively assumed that the target population has an increased risk of dementia compared to the general population, and vary this relative risk from 2.0 to 4.0. The benefits from the dBHS intervention were calculated as offsets in medical costs, non-medical costs, costs of informal care and QALYs gained. To calculate the overall monetary benefit, we have placed the value of a QALY at 30,000 EUR, a conservative estimate close to the annual gross domestic product (GDP) per capita in the EU in 2020 [78,79].

### 4.2 Results from the simulations

**Fig. 2:**
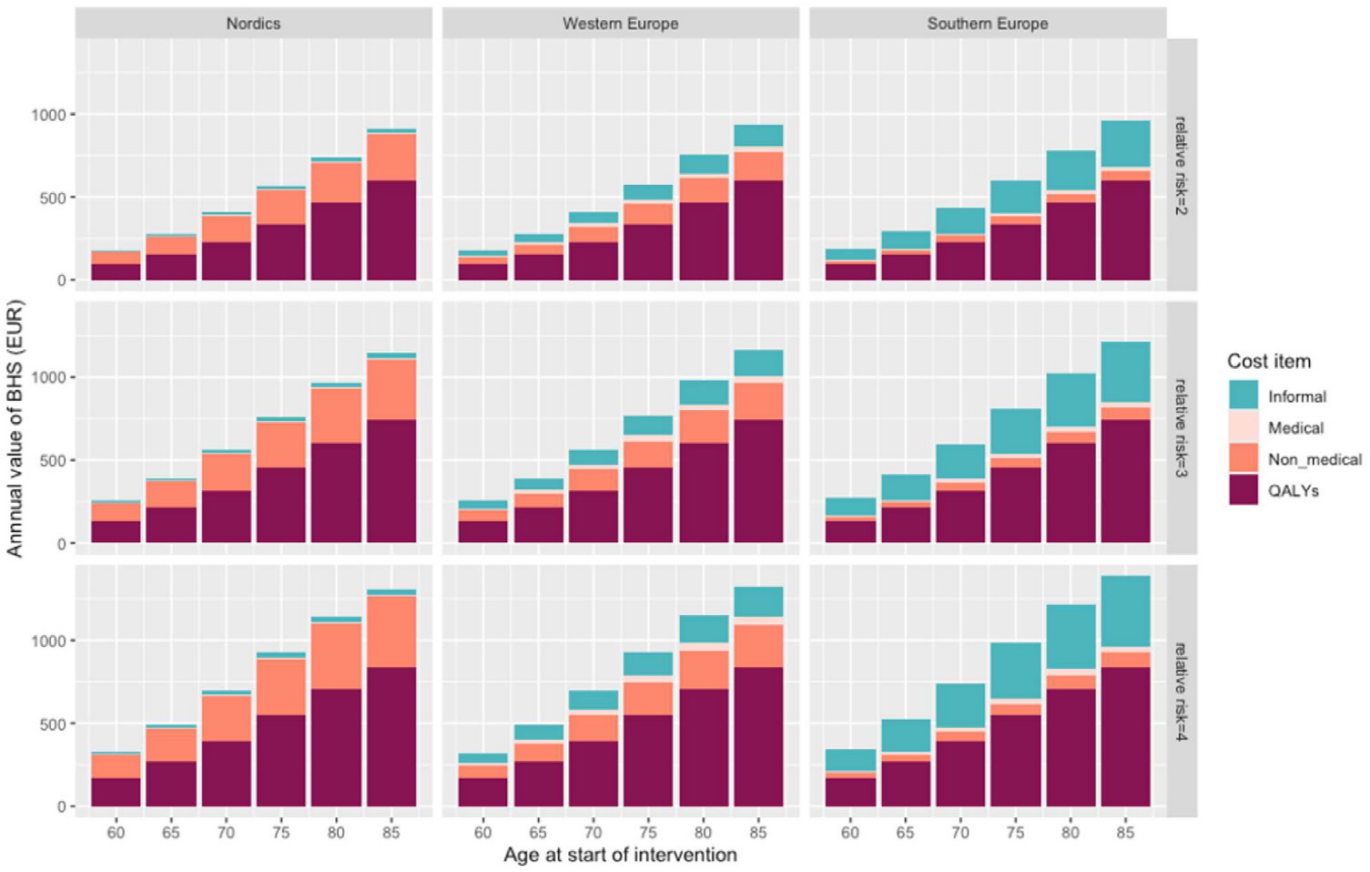
Results from simulations of a hypothetical intervention reducing the risk of dementia by 20% over five years. The figure shows the cost savings and value of health gains (in terms of quality-adjusted life-years, QALYs), expressed as EUR per patient per year of treatment. Results are shown separately by European region and patient group: age at the start of intervention, and relative risk of dementia.

These are presented in Fig. 2 and shown as the annualized value of the dBHS intervention. For a 70-year-old person with a relative risk of dementia of 3, the value of the dBHS intervention is just over 500 EUR per year of intervention. Put differently, the highest price at which the intervention would be cost-effective is just over 500 EUR per year. By comparison, the cost of the two-year FINGER multi-domain intervention has previously been estimated to 5490 SEK, or approximately 500 EUR [75].

Across all regions, QALYs gains represents the most important value driver of dBHS, followed by a reduction in costs of non-medical care and informal care. The relative importance of formal non-medical care and informal care varies by geographic region, with formal institutional care being more predominant in Northern Europe. Offsets in direct medical costs were very small across all regions. The value of the intervention increases for patients in higher risk groups, based on age and relative risk. Clearly, higher risks and higher regional GDP entail higher costs for cost-effective interventions.

There is considerable uncertainty associated with these preliminary estimates. A central assumption is that intervention leads to an indirect reduction in mortality as a result from lower dementia rates; this effect is behind a large share of the QALY gains. If mortality benefits were smaller, QALY gains would be more modest. However, this would be coupled with greater reductions in formal and informal care costs. Further, our estimates do not consider other potentially beneficial health consequences of preventive interventions beyond dementia risk reduction. For example, the FINGER studies have demonstrated to reduce the risk of cerebrovascular events, as well as the risk for new cardiovascular disease (CVD) events in patients with existing CVD [80]. These effects may be substantial and should be considered in future economic evaluations of dBHS.

Beyond the value of reduced dementia risk, there may also be an intrinsic value of improved risk assessment and risk communication. Individuals can have a positive willingness to pay for information on their risk of developing Alzheimer's disease, even when there is no curative treatment [81]. Estimating this value more precisely is challenging both due to limited data availability and methodological issues. No data is currently available regarding the potential health economic effects of cognitive enhancement.

The costs for operating dBHS include the costs for health care visits, examinations, and procedures for diagnosis and risk assessment, non-pharmacological and pharmacological interventions, and follow-up visits. These costs will be visible for health care systems, incurred upfront, and will be comparatively easy to estimate. By contrast, the benefits from dBHS will occur later in time, they will mainly be incurred outside the health care system and appear on no budget or scorecard. Estimating the long-term benefits from dBHS requires projections that are associated with considerable uncertainty, and key stakeholders such as health care payers may not fully consider these effects in their decision making. New payment models may be needed to overcome these hurdles for cost-effective implementation of dBHS [82]. As an example, health impact bonds are financial instruments that extend pay-for-results models by enabling public payers to pay investors retrospectively for health benefits or cost savings after they have been demonstrated [83]. The proceeds from issuing the health impact bonds are used to implement the innovation and setting up a system for monitoring outcomes, while the investors are repaid based on the actual health outcomes and/or economic results. This way financial markets can be leveraged to obtain funding and manage risks.

## 5 Conclusions

dBHS are an innovative health offer aiming to extend healthy life free of cognitive impairment by implementing personalized programs of secondary prevention based on available scientific evidence. Building on the insight from pilot experiences and the discussions held during the conference, the European Task Force for dBHS has identified strategic research priorities to advance the field and ensure the successful implementation and sustainability of dBHS:

• Scaling and standardizing pilot experiences: encourage further collaboration between pilot centers to refine and harmonize protocols for dBHS across diverse healthcare systems. This would facilitate cross-center fertilization and help establish best practices;
• Refining risk estimates for cognitive impairment and dementia that take into account among others age at baseline, time and duration of exposure, genetic and biological risk factors, commonality of risk factors;
• Testing innovative preventive interventions: expand research to evaluate the efficacy of biological interventions targeting mechanisms related to AD pathology including inflammation, and synergy with lifestyle-based approaches;
• Strengthening evidence on cognitive enhancement interventions: prioritize high-quality pharmacological and non-pharmacological (e.g. brain stimulation) clinical trials and their integration into multimodal prevention programs;
• Demonstrating the efficacy of the dBHS patient journey: establish long-term follow-up studies to assess the real-world impact of dBHS on cognitive impairment incidence, cognitive health, and patient-centered outcomes (e.g. quality of life,functional independence, subjective cognitive well-being, satisfaction with care, alignment with personal health goals, etc.);
• Estimating the economic sustainability of dBHS: carry out detailed cost-effectiveness analyses of dBHS in different healthcare contexts, with attention to equity of resource allocation and access;
• Addressing ethical and social implications: investigate the ethical, psychological, and social implications of comprehensive risk communication of genetic, biological and lifestyle risk factors and secondary prevention programs; developing culturally sensitive and inclusive approaches to ensure equitable access to dBHS.

## Funding sources

The event where the ideas of this paper were first presented (International conference and workshop on Brain Health Services for the Prevention of Dementia, February 8 2024, Geneva, Switzerland) was funded by the Swiss National Science Foundation (IZSEZ0_218,815) and unrestricted grants by Biogen, Lilly, Novo Nordisk, OM Pharma, Schwabe Pharma AG. Funders played no role in the writing and submission of this manuscript.

## Stakeholders

The following stakeholders took part to the International conference and workshop on Brain Health Services for the Prevention of Dementia, February 8 2024, Geneva, Switzerland. Scientific societies (delegates are in parentheses): European Academy of Neurology – EAN (Elena Moro, Kristian Steen Frederiksen), Federation of the European Societies of Neuropsychology – FESN (Lisa Cipolotti, Stefano Cappa), European geriatric medicine society – EuGMS (Dorota Religa, Susan D. Shenkin), European Association of Nuclear Medicine EANM (Valentina Garibotto), European Society of Neuroradiology – ESNR (Meike W. Vernooij), Swiss Neurological Society – SNS (Hans Pihan), International Federation of Clinical Chemistry and Laboratory Medicine - IFCCLM (Kaj Blennow), Italian Neurological Society – SIN (Alessandro Padovani). Disease-based professional networks: European FTD network – EFTDN (Barbara Borroni), Swiss Memory Clinics – SMC (Rafael Meyer). Pharma industry: European Federation of Pharma Industry Association (Martin Pan). Private research funders: Synapsis Foundation, Zurich, Switzerland (Margrit Leuthold); Leenaards Foundation, Lausanne (Philippe Moreillon), Switzerland; Association for Research on Alzheimer's – APRA (Tim Brockmann), Geneva, Switzerland. Patient advocates: Alzheimer Europe, Brain Health Scotland, Alzheimer Switzerland (Stefanie Becker), Alzheimer Genève (Sophie Courvoisier). Charities and international organizations: World Dementia Council (Philip Scheltens).

## CRediT authorship contribution statement

**Giovanni B. Frisoni:** Writing – original draft. **Federica Ribaldi:** Writing – original draft. **Gilles Allali:** Writing – review & editing, Conceptualization. **Théophile Bieth:** Writing – review & editing. **Andrea Brioschi Guevara:** Writing – review & editing. **Stefano Cappa:** Writing – review & editing. **Lisa Cipolotti:** Writing – review & editing. **Kristian Steen Frederiksen:** Writing – review & editing. **Jean Georges:** Writing – review & editing. **Frank Jessen:** Writing – review & editing. **Giacomo Koch:** Writing – review & editing. **Hugh Masters:** Writing – review & editing. **Augusto J. Mendes:** Writing – review & editing. **Lutz Frölich:** Writing – review & editing. **Valentina Garibotto:** Writing – review & editing. **Oriol Grau-Rivera:** Writing – review & editing. **Federico E. Pozzi:** Writing – review & editing. **Dorota Religa:** Writing – review & editing. **Ayda Rostamzadeh:** Writing – review & editing. **Lenny Shallcross:** Writing – review & editing. **Susan D. Shenkin:** Writing – review & editing. **Wiesje M. van der Flier:** Writing – review & editing. **Meike W. Vernooij:** Writing – review & editing. **Leonie N.C. Visser:** Writing – review & editing. **Jeffrey L. Cummings:** Writing – review & editing. **Philip Scheltens:** Writing – review & editing. **Bruno Dubois:** Writing – review & editing. **Elena Moro:** Writing – review & editing. **Claudio L.A. Bassetti:** Conceptualization. **Miia Kivipelto:** Writing – review & editing.

## Declaration of competing interest

Giovanni B. Frisoni has received funding through the Private Foundation of Geneva University Hospitals from: A.P.R.A. – Association Suisse pour la Recherche sur la Maladie d'Alzheimer, Genève; Fondation Segré, Genève; Ivan Pictet, Genève; Race Against Dementia Foundation, London, UK; Fondation Child Care, Genève; Fondation Edmond J. Safra, Genève; Fondation Minkoff, Genève; Fondazione Agusta, Lugano; McCall Macbain Foundation, Canada; Nicole et René Keller, Genève; Fondation AETAS, Genève. GBF has received funding through the University of Geneva or Geneva University Hospitals: for IISSs from ROCHE Pharmaceuticals, OM Pharma, EISAI Pharmaceuticals, Biogen Pharmaceuticals and Novo Nordisk; for competitive research projects from: H2020, Innovative Medicines Initiative (IMI), IMI2, Swiss National Science Foundation, and VELUX Foundation; for consulting from: Biogen, Diadem, Novo Nordisk, and Roche; for honoraria for lectures, presentations, speakers bureaus, manuscript writing, or educational events from: Biogen, Roche, Novo Nordisk, and GE HealthCare.

Federica Ribaldi is funded in part by the Swiss National Science Foundation under grant agreement 320,030_182,772: Brain connectivity and metacognition in persons with subjective cognitive decline (COSCODE).

Gilles Allali is supported by the Swiss National Science Foundation (grant# 214,855), the Leenaards Foundation, the Solis Foundation, the Empiris Foundation and the Synapsis Foundation. GA serves as a scientific advisor for Roche and Lilly and received speaker's fees from Schwabe and Lilly.

Andrea Brioschi Guevara has received funding from the Swiss Health Promotion foundation, Leenaards foundation, Dragon Bleu and Floshield foundations.

Kristian Steen Frederiksen serves on an advisory board for Novo Nordisk and Eisai AD with compensation paid to his institution. KSF has been an invited speaker for Novo Nordisk and Lundbeck A/S with compensation paid to his institution. KSF has served as associate editor since 2023 and is the incoming Editor-in-Chief for Alzheimer´s Research & Therapy.

Jean Georges has received funding for the EURO-FINGERS project which is supported through the following funding organisations under the aegis of JPND www.jpnd.eu: Finland, Academy of Finland; Germany, Federal Ministry of Education and Research; Spain, National Institute of Health Carlos III; Luxembourg, National Research Fund; Hungary, National Research, Development and Innovation Office; Netherlands, Netherlands Organisation for Health Research and Development; Sweden, Swedish Research Council. Grant agreement: INTER/JPND/19/BM/14,012,609.

Frank Jessen has received fees for advisory boards and speaker fees from: AC immune, Biogen, Cogthera, Eisai, Eli Lilly, GE Healthcare, Grifols, Janssen, Novo Nordisk, Roche.

Giacomo Koch has received funding (competitive grants) not related to the current manuscript from the Alzheimer Drug Discovery Foundation (ADDF), European Commission Horizon 2020, Italian Ministry Of Health, Italian Ministry of Education (MIUR), Brightfocus Foundation. GK also received funding from PIAM farmaceutici Spa and Epitech Group. GK is scientific co-founder and holds stocks of Sinaptica Therapeutics. GK has received payment or honoraria for lectures, presentations, speakers bureaus, manuscript writing, or educational events from: Epitech, Roche, Novo Nordisk. GK has a patent on “combination drug formulations including rotigotine and an acetylcholinesterase inhibitor for the treatment of neurodegenerative diseases” (20,220,040,148) and another on “systems and methods for providing personalized targeted non-invasive stimulation to a brain network” (20,230,381,512).

Lutz Frölich is an investigator in clinical trials sponsored by Axon Neuroscience, Anavex, Alector, Boehringer Ingelheim, Eisai, Hummingbird, NovoNordisk, Noselab and serves on an advisory board for Biogen, BioVie, Eisai, Grifols, Janssen Cilag, Neurimmune, Noselab, NovoNordisk, Roche, TauRX, Schwabe with personal compensation. LF has received honoraria for Clinical Study Committees from Avanir/Otsuka, PharmatrophiX, Charité Berlin, Neuroscios, Vivoryon.

Valentina Garibotto is supported by the Swiss national science foundation (project n.320030_185,028 and 320,030_169,876), the Aetas Foundation, the Schmidheiny Foundation, the Velux Foundation, the Synapsis foundation, the Fondation privée des HUG. VG received support for research and speakers‘fees from Siemens Healthineers, GE HealthCare, Janssen, Novo Nordisk, all paid to her institution.

Oriol Grau-Rivera is supported by the Spanish Ministry of Science, Innovation and Universities (IJC2020–043,417-I), receives research support from F. Hoffmann-La Roche Ltd and has given lectures in symposia sponsored by Roche Diagnostics.

Ayda Rostamzadeh has received fees from Eisai for presentations.

Wiesje M. van der Flier has been funded by ZonMW, NWO, EU-FP7, EU-JPND, Alzheimer Nederland, Hersenstichting CardioVascular Onderzoek Nederland, Health Holland, Topsector Life Sciences & Health, stichting Dioraphte, Gieskes-Strijbis fonds, stichting Equilibrio, Edwin Bouw fonds, Pasman stichting, stichting Alzheimer & Neuropsychiatrie Foundation, Philips, Biogen MA Inc, Novartis-NL, Life-MI, AVID, Roche BV, Fujifilm, Combinostics. WvdF holds the Pasman chair. WvdF has performed contract research for Biogen MA Inc, and Boehringer Ingelheim. WvdF has been an invited speaker at Boehringer Ingelheim, Biogen MA Inc, DaThe authors declare that they have no known competing financial interests or personal relationships that could have appeared to influence the work reported in this paper.
